# Role of ATP in Extracellular Vesicle Biogenesis and Dynamics

**DOI:** 10.3389/fphar.2021.654023

**Published:** 2021-03-15

**Authors:** Marta Lombardi, Martina Gabrielli, Elena Adinolfi, Claudia Verderio

**Affiliations:** ^1^ CNR Institute of Neuroscience, Research Labs–University Milano-Bicocca, Vedano al Lambro, Italy; ^2^ Department of Medical Sciences, Section of Experimental Medicine, University of Ferrara, Ferrara, Italy

**Keywords:** ATP, extracellular vesicles, immune cells, tumor cells, P2X7 receptor, extracellular vesicle biogenesis, extracellular vesicle morphology, ATP cargo

## Abstract

Adenosine triphosphate (ATP) is among the molecules involved in the immune response. It acts as danger signal that promotes inflammation by activating both P2X and P2Y purinergic receptors expressed in immune cells, including microglia, and tumor cells. One of the most important receptors implicated in ATP-induced inflammation is P2X7 receptor (P2X7R). The stimulation of P2X7R by high concentration of ATP results in cell proliferation, inflammasome activation and shedding of extracellular vesicles (EVs). EVs are membrane structures released by all cells, which contain a selection of donor cell components, including proteins, lipids, RNA and ATP itself, and are able to transfer these molecules to target cells. ATP stimulation not only promotes EV production from microglia but also influences EV composition and signaling to the environment. In the present review, we will discuss the current knowledge on the role of ATP in the biogenesis and dynamics of EVs, which exert important functions in physiology and pathophysiology.

## Introduction

Adenosine triphosphate (ATP) is a ubiquitous nucleotide that not only provides energy source within cells but acts as transmitter/signaling molecule mediating interactions among various cell types in the brain (Inoue, 2002; Hansson and Ronnback, 2003) and many other organs and systems.

Under physiological conditions, the concentration of extracellular ATP (eATP) is very low (400–1,000 nM), allowing a 10^6^-fold gradient for ATP efflux (Trautmann, 2009). Larger increase in eATP levels occurs during metabolic stress or brain injury, and persists in the peritraumatic zone for many hours after the insult (Wang et al., 2004). Indeed, at the site of injury activated immune cells, i.e., lymphocytes (Filippini et al., 1990), macrophages (Sikora et al., 1999), microglia (Ferrari et al., 1997), and platelets (Beigi et al., 1999), release ATP and other purines, such as adenosine diphosphate (ADP) and uridine triphosphate (UTP) into the extracellular space mainly via exocytosis of secretory granules or transport through channels or transporters (Lazarowski, 2012).

eATP acts as a Danger Associated Molecular Patterns (DAMPs) and binds to specific surface receptors called P2 purinoceptors, promoting acute inflammation (Soni et al., 2019). There are two subsets of P2 receptors: P2Y and P2X receptors (Idzko et al., 2014). P2Y receptors (P2YR) are G-protein-coupled receptors, which mediate adenylyl cyclase, phospholipase C and ion channel activation (Abbracchio et al., 2006). On the contrary, P2X receptors (P2XR) are Ca^2+^-permeable, non-selective cation channels sensitive to micromolar concentration of eATP (Trautmann, 2009). Both P2YR and P2XR are expressed on microglia, the immune cells resident in the brain, along with receptors specific for the ATP metabolite Adenosine, and are necessary for the rapid microglial response to changes in brain homeostasis (Orr et al., 2009; Illes et al., 2020).

During inflammation, microglia undergo progressive modifications, including altered expression of cell surface markers and inflammation-related genes, process retraction and acquisition of an ameboid morphology, enhanced migration and phagocytic ability (Kettenmann et al., 2011). These changes in microglial functions are partly associated with changes in purinergic receptors expression that determine different responses to ATP. Process retraction is mainly due to upregulation of adenosine receptor A2A and downregulation of P2Y12 receptors (Orr et al., 2009), whereas migration is mediated by adenosine A1 and P2X4 (Li et al., 2013) as well as P2Y12 receptors (Haynes et al., 2006; Ohsawa et al., 2007). Phagocytosis is triggered by the upregulation of P2Y6R, which is activated by the release of UTP by dying cells (Inoue, 2007). Finally, the ATP-sensitive P2X7 receptor (P2X7R) has been shown to drive important morphological alterations in microglia as well as the release of pro-inflammatory/pathological agents via extracellular vesicles (EVs) (Ferrari et al., 2006).

The present review focuses on the role of ATP/P2X7R signaling axis in inducing EV shedding from immune and tumor cells, and on ATP involvement in the control of EV composition and dynamics of interaction with target cells.

## ATP Stimulates the Release of EVs by Immune Cells Upon P2X7R Activation

P2X7 receptor (P2X7R) is highly expressed on inflammatory cells (Faas et al., 2017) and requires a very high concentration (>100 µM) of ATP for its activation (Trautmann, 2009). Once stimulated, influx of Na^+^ and Ca^2+^ into the cell and efflux of K^+^ out of the cell occur, inducing cell proliferation (Nuttle and Dubyak, 1994; Bianco et al., 2006) and inflammasome activation (Yaron et al., 2015; Orioli et al., 2017). Furthermore, upon prolonged activation, P2X7R forms an aqueous pore at the cell membrane allowing the passage of hydrophilic molecules, that results in cell death (Faas et al., 2017).

Fifteen years ago, Verderio and colleagues demonstrated another fundamental function mediated by P2X7R activation in cultured microglia. P2X7R stimulation massively increases the shedding of large membrane vesicles from the plasma membrane (PM). These large extracellular vesicles (EVs), also known as microvesicles, are circular membrane structures enriched in bioactive molecules that play an important role in cell-to-cell communication (Bianco et al., 2009).

**FIGURE 1:**
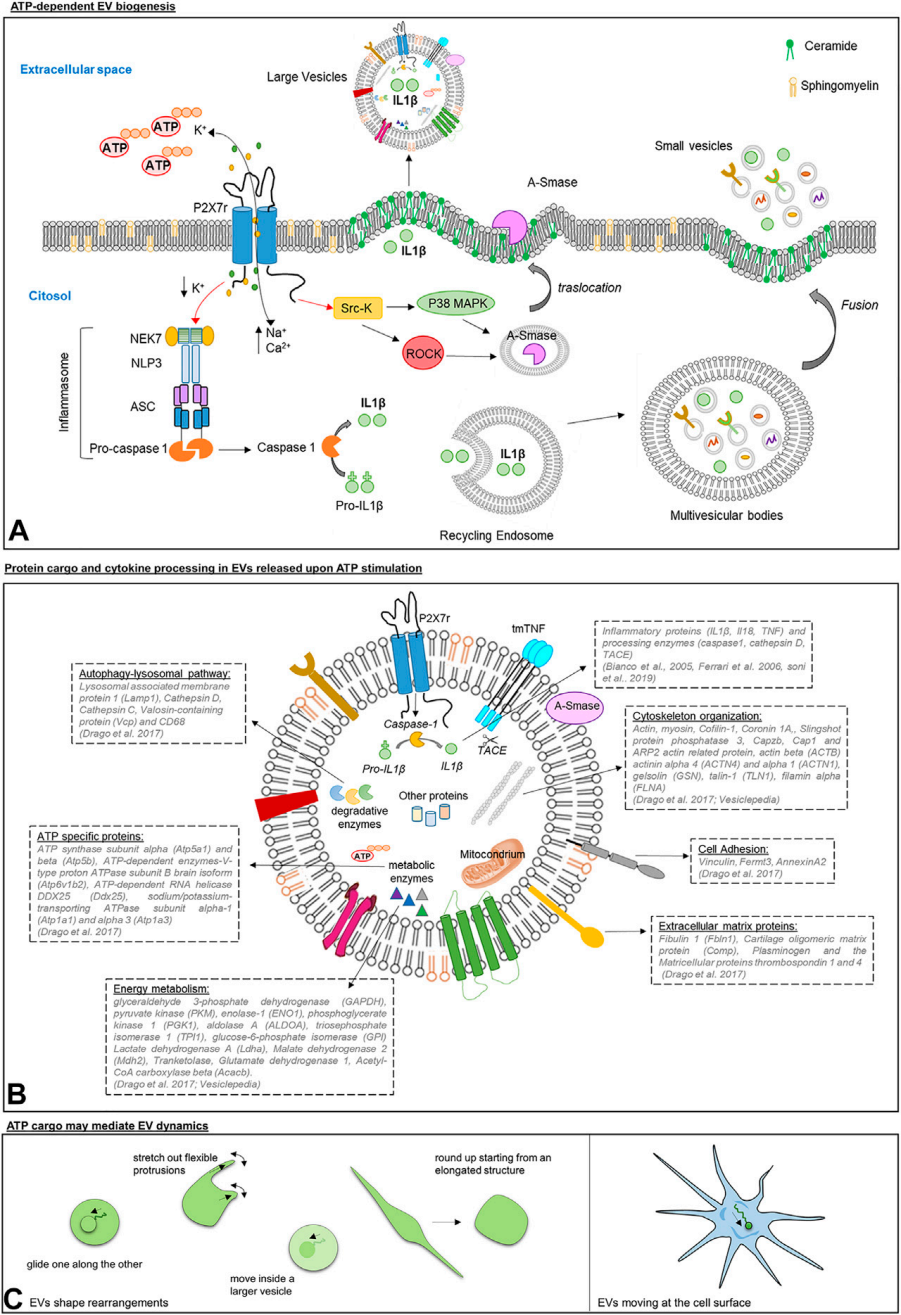
Panel **(A)**: Scheme of ATP/P2X7R signaling axis inducing EV shedding from immune cells. Upon ATP stimulation, P2X7R activates *p*38 MAPK and ROCK through Src kinases. In turn, *p*38 and ROCK trigger the local disassembly of the cytoskeletal elements and the mobilization of A-SMASE from the luminal lysosomal compartment to the outer leaflet of the PM, where the enzyme hydrolyzes sphingomyelin to ceramide favoring blebs formation and the shedding of large vesicles carrying IL-1β (Bianco et al., 2005).P2X7R also regulates the release of small EVs. ATP-induced P2X7R stimulation drives the assembly and the activation of the inflammasome composed by regulatory proteins, NEK7, ASC and NLRP3, which are essential for caspase-1 activation. Caspase 1 is a protease implicated in IL-1β processing and in regulating the membrane trafficking pathways that control multivesicular bodies fusion with the PM and the release of IL-1β storing small EVs (Qu et al., 2009). Panel **(B)**: Schematic representation of protein cargo and cytokine processing in large EVs released upon ATP stimulation. By activating P2X7R, ATP induces in large EVs the processing of inflammatory cytokines (Hide et al., 2000; Bianco et al., 2005; Ferrari et al., 2006) and sorting of proteins implicated in autophagy-lysosomal pathway, phagocytosis and endocytosis, energy metabolism and cell adhesion/extracellular matrix organization (Drago et al., 2017). Panel **(C)**: Graphic representation of morphological changes of EVs isolated from human mast cell lines, human blood serum, mouse lung, and *Saccharomyces* cerevisiae as imaged by Cvjetkovic and colleagues (Cvjetkovic et al., 2017) **(left)**, and of a single EV in motion at the cell surface of microglia (Prada et al., 2016) **(right)**.

Differently from other members of the P2X family, P2X7R present a long cytoplasmic C terminus that contains several binding sites for Src kinases proteins, which phosphorylate and activate ROCK and p38 MAP kinases (Kanthou and Tozer, 2002; Pfeiffer et al., 2004). These signaling proteins induce the local disassembly of the cytoskeletal elements and the translocation to the PM of the enzyme acid sphigomyelinase (A-SMASE). A-SMASE hydrolyzes sphingomyelin, a phospholipid abundant in the outer leaflet of the PM, to ceramide, facilitating blebs formation and EV shedding (Figure 1A) (Bianco et al., 2009).

Notably, surface blebbing occurs in proximity of lipid rafts (Del Conde et al., 2005), where P2X7R localizes, and requires the loss of membrane asymmetry and the exposure of phosphatidylserine at the outer leaflet of PM. Vesicle shedding causes a decrease in PM capacitance (MacKenzie et al., 2001) and is markedly inhibited by removal of extracellular Ca^2+^ or treatment with either P2X7R antagonists (Bianco et al., 2005; Pizzirani et al., 2007) or p38 and rho kinases inhibitors (Pfeiffer et al., 2004). In accordance, membrane blebbing is increased by antagonism of the P2X7R negative regulator HSP90 (Adinolfi et al., 2003).

In addition to large EVs, P2X7R stimulation triggers release of small EVs, also called exosomes, originating in the endocytic compartment (Figure 1 Panel A) (Asai et al., 2015; Ruan et al., 2020).

The major finding related to P2X7R-dependent EV production from microglia and peripheral immune cells is linked to its involvement in the processing and release of inflammatory cytokines (MacKenzie et al., 2001; Bianco et al., 2005).

Several lines of evidence indicated that EVs are loaded with unprocessed pro-IL-1β, mature IL-1β and the IL-1β converting enzyme caspase-1, and express P2X7R in their membranes (Bianco et al., 2005; Pizzirani et al., 2007). Caspase-1 is activated upon P2X7R stimulation on the vesicle surface, and is responsible for conversion of the biological inactive IL-1β precursor into the active form of the cytokine (Bianco et al., 2005) (Figures 1A,B). Other reports showed that EVs act as carriers of the protease cathepsin D (Qu et al., 2009; Sarkar et al., 2009) besides caspase-1, and other cytokines such as TNF and IL-18 (Hide et al., 2000; Ferrari et al., 2006).

Specifically, Barbera-Cremades and colleagues showed that stimulation of P2X7R in macrophages leads to the release of EVs containing both TNF and the TNF-converting enzyme (TACE), that cleaves membrane-bound TNF, generating the soluble cytokine (Barbera-Cremades et al., 2017; Raffaele et al., 2020). Furthermore, a recent study demonstrated that ATP redirects TNF intracellular trafficking in activated macrophages, limiting the release of soluble TNF and preferentially packaging transmembrane TNF in EVs (Soni et al., 2019). Importantly, TNF-carrying EVs are biologically more potent than soluble TNF, and mediate significant lung inflammation in mice (Soni et al., 2019).

Through EV production, inflammatory proteins can be released at significant distance from donor cell, in possible proximity to target cells, thus preventing the dispersal and degradation of mediators in the extracellular environment.

In recent years, several studies investigated the physiological and pathological functions of EVs within the brain. These functions include control of neuronal development (Marzesco et al., 2005), synaptic activity (Antonucci et al., 2012; Gabrielli et al., 2015), axon-glial transfer of information (Prada et al., 2018), nerve regeneration (Lai and Breakefield, 2012) and myelin formation (Pusic et al., 2016; Van Niel et al., 2018; Lombardi et al., 2019); as well as disease-associated events, such as tumor progression, spreading of inflammation (Verderio et al., 2012) or dissemination of pathogenic proteins (Joshi et al., 2014; Asai et al., 2015; Eitan et al., 2016; Sardar Sinha et al., 2018; Crotti et al., 2019; Ruan et al., 2020). However, the contribution of ATP-induced EV shedding vs. constitutive EV release in brain disease pathogenesis is just emerging.

ATP-induced EVs may play a relevant pathogenic role in traumatic brain injury (TBI). After TBI, high concentrations of eATP activate P2X7R in microglia and increase EV production, while treatment of TBI-affecting rats with the P2X7R antagonist A804598 or the immune modulator FTY720, that inhibits A-SMase-dependent EV biogenesis (Verderio et al., 2012), significantly decreases the number of microglial EVs in the injured/adjacent regions and in cerebrospinal fluid (CSF), and improves disease outcome (Liu et al., 2017).

Another study implicated microglial EVs released upon ATP stimulation of P2X7R in the spreading of tau protein and disease progression in a tauopathy mouse model. Specifically, pharmacologic blockade of P2X7R with GSK1482160, an orally applicable and CNS-penetrant inhibitor, suppressed both secretion of small EVs (exosomes) and disease outcome in the early disease stages (Ruan et al., 2020).

Conversely, the role of ATP and P2X7R-mediated EV release in multiple sclerosis (MS), the prototypical neuroinflammatory disease, remains controversial. Treatment of EAE mice, a MS mouse model, with the specific P2X7R antagonists oxATP and BBG reduced disease severity (Matute et al., 2007), but P2X7R knockout mice displayed a more severe pathology (Chen and Brosnan, 2006). Furthermore, EAE ameliorated in A-SMASE knock-out mice, genetically impaired in ATP-evoked EV production (Verderio et al., 2012), but injection of the A-SMASE inhibitor imipramine did not significantly reduce the level of myeloid EVs in the CSF. The latter finding rules out a major role of ATP in sustaining EV production in a context of chronic neuroinflammation, where cytokines may mainly control EV release from myeloid cells (Colombo et al., 2018).

## P2X7R-Dependent EV Production From Tumor Cells

The tumor microenvironment is rich in eATP, and the role of this nucleotide and its receptors, particularly P2X7R, in cancer has been the focus of numerous papers in recent years (Di Virgilio et al., 2018; Adinolfi et al., 2019; Lara et al., 2020). P2X7R is upregulated in solid cancer and onco-hematological conditions, and several preclinical studies have demonstrated that its blockade has good potential as an anticancer treatment (Adinolfi et al., 2012; De Marchi et al., 2019; Pegoraro et al., 2020). Recently, the association of P2X7R with EV release has been supported by a work showing an increase in cancer patient's serum concentration of soluble P2X7R, possibly expressed on the surface of EVs (Giuliani et al., 2019). As mentioned above, the activation of P2X7R is also associated with the release of EVs from the monocyte/macrophage cell lineage (Baroni et al., 2007; Pizzirani et al., 2007; Gulinelli et al., 2012). These vesicles carry several molecules, including cytokines and tissue factor, associated with cancer pathogenesis and progression but also with tumor immune eradication and immunosuppression (Graner, 2018). EVs released from both cancer and immune cells have shown to facilitate angiogenesis, cause extracellular matrix remodeling, prepare the pre-metastatic niche, and consequently cause organ tropism of disseminating tumor cells (Kuriyama et al., 2020; Schubert and Boutros, 2021). However, only few manuscripts have reported P2X7-dependent EV release from cancer cells (Gutierrez-Martin et al., 2011; Kholia et al., 2015; Park et al., 2019) and therefore evidence relating to P2X7R activity, EV content, and cancer function is far to be complete and will deserve further attention.

## ATP Stimulation Influences EV Composition

So far, only one study showed that ATP strongly influences the composition of EVs (Drago et al., 2017). Label free proteomics revealed that ATP stimulation induces sorting into microglial EVs of proteins implicated in autophagy-lysosomal pathway, phagocytosis and endocytosis, energy metabolism and cell adhesion/extracellular matrix organization (Drago et al., 2017).

The overexpression of degradative enzymes in EVs produced by ATP-stimulated microglia (ATP-EVs), compared to those released constitutively, may reflect the enhanced capacity of microglia to phagocyte apoptotic cells or synapses in response to ATP. By contrast, the abundance of metabolic enzymes necessary for glycolysis, lactate production, the oxidative branch of the pentose phosphate pathway, glutamine metabolism and fatty acid synthesis may reflect an increase in cellular metabolism to sustain ATP-dependent microglial functions, such as process scanning and phagocytic activity (Grabert et al., 2016).

Due to the higher content of proteins involved in extracellular matrix organization and cell adhesion, ATP-EVs adhere more and have stronger capacity to activate cultured astrocytes compared to constitutive EVs.

Collectively, these data indicate that ATP stimulation not only promotes EV production from microglia but also enhances their signaling to the environment.

## ATP is Among the Cargo of EVs

ATP is a component of EVs (Graner, 2018). A pioneer study by Ronquist and colleagues showed that small EVs generated in the endocytic compartment of prostate epithelial cells, also called prostasomes, can produce ATP by glycolysis. Prosteasomes contain glycolytic enzymes and their capacity to produce ATP from fructose or glucose has been proven by the luciferin/luciferase assay (Ronquist et al., 2013). Interestingly, glycolytic enzymes, generating ATP from glucose, have been systematically reported in EVs of different cell origin, including EVs produced by mesenchymal stem cells, which have the capacity to restore ATP levels when delivered to an ischemic tissue (Arslan et al., 2013). Among glycolytic enzymes, 3-phosphate dehydrogenase (GAPDH), pyruvate kinase (PKM), enolase-1 (ENO1), phosphoglycerate kinase 1 (PGK1), aldolase A (ALDOA), triosephosphate isomerase 1 (TPI1) and glucose-6-phosphate isomerase (GPI) are listed among the 100 proteins more often identified in EVs according to the database Vesiclepedia (http://microvesicles.org/), suggesting that ATP production by glycolysis may be a common feature of EVs (Figure 1B).

Furthermore, it has been recently shown that mitochondria, the main source of cellular ATP, can be also packaged into EVs (Figure 1B) (Hough et al., 2018; Zhang et al., 2020), further indicating that ATP can be generated in metabolically active EVs.

## ATP Cargo may Influence the Dynamics of EV Interaction With Recipient Cells

Inside EVs ATP may represent a crucial source of energy, able to fuel active processes, such as the activity of ATP-dependent enzymes, e.g., V-type proton ATPase subunit B (Atp6v1b2), RNA helicase DDX25 (Ddx25), Sodium/potassium-transporting ATPase subunit alpha-1 and 3 (Atp1a1, Atp1a3), which are part of the proteome of microglial EVs (Figure 1B) (Drago et al., 2017). More importantly, vesicular ATP may support cytoskeleton rearrangements. Consistently, a large body of evidence has located *actin* inside EVs, a key component of the cellular cytoskeleton mediating cell migration and shape changes, and allowing cells to form adhesion with each other and with the extracellular matrix (Figures 1A,C) (Svitkina, 2018). Cryo-electron micrographs imaged actin-like filaments in a subpopulation of EVs isolated from different biological samples [fresh plasma, (Yuana et al., 2013); human ejaculate, (Hoog and Lotvall, 2015); human ejaculate and human mast cell cultures, (Cvjetkovic et al., 2017); HeLa cells, (Yang et al., 2020)] and its presence was confirmed by mass-spectrometry, western blot analysis or mRNA Microarray in EVs from most cell types (human dendritic cells, (Kowal et al., 2016); mouse microglia, (Drago et al., 2017); mesenchymal stem cells, (Adamo et al., 2019); HeLa cells, (Yang et al., 2020); osteoclasts, (Holliday et al., 2019); human blood, (Eguchi et al., 2020), and more), together with actin-binding proteins and regulators of actin cytoskeleton. Not surprisingly, actin beta (ACTB) and several actin network proteins (such as actinin alpha 4 (ACTN4) and alpha 1 (ACTN1), gelsolin (GSN), cofilin-1 (CFL1), talin-1 (TLN1), filamin alpha (FLNA)) are among the top 100 proteins more frequently detected in EVs on Vesiclepedia (Figure 1B Panel B). These data open up the fascinating possibility that EVs, exploiting actin complexes present in their lumen and ATP as energy source, may have an intrinsic capacity to change their shape to interact with target cells.

In support to this hypothesis, findings from Jan Lötvall’s and Johanna L Höög’s laboratories have shown that, among EVs either isolated from cell cultures, biological fluids or tissue, a part (albeit small) displays morphological changes detectable by time-lapse fluorescence imaging Cvjetkovic et al., 2017. EVs could round up starting from an elongated structure, glide one along the other, move inside a larger vesicle and, importantly, stretch out flexible protrusions (Figure 1C). Being exhibited also by EVs from *Saccharomyces cerevisiae,* these phenomena seem to be evolutionary conserved. Furthermore, time-lapse imaging revealed that single EVs, produced by microglia and gently placed in contact with other microglial cells by optical manipulation, can move after adhesion along the cell surface toward sites of internalization (Figure 1C) (Prada et al., 2016). These findings point at the possibility for EVs to undergo an ATP-dependent actin-mediated form of extracellular motion. Further experiments will be necessary to verify this captivating hypothesis. Intriguingly, intrinsic active motility would allow EVs to travel in the extracellular space at the cell surface, independently from fluid fluxes or cell-driven mechanisms (e.g., filopodia surfing/grabbing/pushing, (Heusermann et al., 2016), and we can speculate that it might even ease cell entry at specific sites of the PM. These perspectives are absolutely worth to be better explored in the future.

## Conclusion

The interest for EVs released upon ATP stimulation has increased exponentially, given that they have become vehicle of inflammatory signals (Verderio et al., 2012), tumorigenic factors (Graner, 2018) or misfolded proteins in neurodegenerative diseases (Ruan et al., 2020), and their number is significantly augmented in the body fluids of patients affected by many neurological diseases (Verderio et al., 2012; Colombo et al., 2018). Since the analysis of EV cargo may provide indications on the activation state of donor cells and the pathological state of the brain, EVs are currently under intense investigation for a possible employment in clinical practice as prognostic biomarkers. In addition, further knowledge of EV dynamics and interaction with target cells may reveal new molecular targets to limit cancer metastasis and propagation of neurodegenerative lesions throughout the brain.

## Abbreviations

ACTB, actin beta; ACTN4, actinin alpha 4; ACTN1, actinin alpha 1; ADP, adenosine diphosphate; ALDOA, aldolase A; A-SMASE, acid sphigomyelinase; ATP, adenosine triphosphate; ATP-EVs, EVs produced by ATP-stimulated microglia; Atp1a1, sodium/potassium-transporting ATPase subunit alpha-1; Atp1a3, sodium/potassium-transporting ATPase subunit alpha-3; Atp5a1, ATP synthase subunit alpha; Atp5b, ATP synthase subunit beta; Atp6v1b2, V-type proton ATPase subunit B brain isoform; CFL-1, cofilin-1; CSF, cerebrospinal fluid; DAMPs, Danger Associated Molecular Patterns; Ddx25, ATP-dependent RNA helicase DDX25; eATP, extracellular ATP; ENO1, enolase-1; FLNA, filamin alpha; GAPDH, glyceraldehyde 3-phosphate dehydrogenase; GPI, glucose-6-phosphate isomerase; GSN, gelsolin; EVs, extracellular vesicles; LDHA, lactate dehydrogenase A; LDHB, lactate dehydrogenase B; MS: multiple sclerosis; PGK1, phosphoglycerate kinase 1; PM, plasma membrane; PKM: pyruvate kinase; P2XR, P2X receptors; P2X7R, P2X7 receptor; P2YR, P2Y receptors; TACE, TNF -converting enzyme; TBI, traumatic brain injury; TLN1, talin-1; TPI1, triosephosphate isomerase 1; UTP, uridine triphosphate
